# Symptoms, symptom severity, and contact with primary health care among nonhospitalized COVID-19 patients: a Norwegian web-based survey

**DOI:** 10.1080/02813432.2023.2266477

**Published:** 2023-11-29

**Authors:** Guro H. Fossum, Anja Maria Brænd, Silje Rebekka Heltveit-Olsen, Guri Rørtveit, Sigurd Høye, Jørund Straand

**Affiliations:** a The Antibiotic Centre for Primary Care, Department of General Practice, University of Oslo, Oslo, Norway; b General Practice Research Unit (AFE), Department of General Practice, University of Oslo, Oslo, Norway; c Department of Global Public Health and Primary Care, University of Bergen, Bergen, Norway; d Research Unit for General Practice, NORCE Norwegian Research Centre, Bergen, Norway

**Keywords:** SARS-CoV-2, COVID-19, primary health care, signs and symptoms, surveys and questionnaires

## Abstract

**Objective:**

Dependent on clinical setting, geography and timing during the pandemic, variable symptoms of COVID-19 have been reported. Our aim was to describe self-reported symptom intensity and contact with primary health care among nonhospitalized COVID-19 patients.

**Design:**

Web-based survey.

**Setting:**

Norway between March 2020 and July 2021.

**Subjects:**

Adults in home isolation.

**Main outcome measures:**

Participants reported possible COVID-19 symptoms, duration of symptoms, score of symptom severity (Likert scale 0–3), risk factors, comorbidity, and questions regarding follow-up and information from primary health care.

**Results:**

Of 477 participants, 379 (79%) had PCR-confirmed COVID-19, 324 (68%) were females, and 90% were younger than 60 years. Most common symptoms were “fatigue and/or muscle ache” (80%), nasal symptoms (79%), and headache (73%). The mean severity of symptoms was generally low. Symptoms with the highest mean scores were “fatigue and/or muscle ache” (1.51, SD 1.02) and headache (1.27 (SD 1.00). Mean scores for severity ranged from 0.28 (nausea) to 1.51 (fatigue and/or muscle ache). Women reported higher symptom scores than men. For “affected sense of smell and/or taste”, patients either reported a high symptom score (24%) or no affliction at all (49%). A third of the participants (32%) were followed-up by primary care health personnel, and almost 40% had sought or received information about COVID-19 from general practitioners.

**Conclusion:**

The mean severity of symptoms among nonhospitalized adult COVID-19 patients was generally low. We found large variations in the occurrence and severity of symptoms between patients.

## Key points

Dependent on clinical setting, geography and timing during the pandemic, variable symptoms, and follow-up of COVID-19 have been reported.

The most common symptoms were fatigue and/or muscle ache, nasal symptoms, and headache, and the overall mean symptom score was low.

The symptom where most patients were severely afflicted was a reduced sense of smell and/or taste.

Patients with a higher symptom severity were more likely to receive information about COVID-19 from their GP.

## Introduction

Early research on coronavirus disease 2019 (COVID-19), caused by severe acute respiratory syndrome coronavirus 2 (SARS-CoV-2), reported symptoms in hospitalized patients to be fever and cough [1]. However, an early population-based report including approximately 44,500 patients with confirmed infection, showed that 81% of patients had none or only mild symptoms [2]. The spectrum of symptomatic infections ranged from mild to critical and fatal, with most cases being mild [2–4]. A Cochrane report from 2022 mentioned the loss of smell and taste, as well as cough and fever, as more characteristic of COVID-19 than classic upper respiratory tract symptoms like sore throat, coryza, and rhinorrhoea [5]. A variety of symptoms have been reported across studies conducted in different settings and during different time periods of the pandemic [5].

In Norway, 95% of patients with RTIs are normally handled in general practice, comprising about 15% of all consultations [6]. After the lockdown in March 2020, follow-up of patients with COVID-19 outside hospitals was organized by the municipalities and varied between them, with nurse- and/or general practitioner-lead (GP) follow-up being the most common [7,8]. Routines for additional follow-up after the initial management varied but were often based on telephone calls or video consultations by the GP or dedicated primary health care personnel, regularly for a given time period, or until clinical recovery. Even though most patients are initially examined in primary care, symptoms, and symptom severity are not often investigated in studies from the primary care setting.

In this study from Norway during the first 15 months of the COVID-19 pandemic, we aim to describe self-reported symptoms among suspected and confirmed nonhospitalized COVID-19 patients and their contact with primary health care during their illness.

## Methods

Participants were recruited through an online survey named *CovidNor – the Patient Study*, accessible through an open website run by the University of Oslo from 29 March 2020 to 31 June 2021. Adults in home isolation or quarantine due to COVID-19 were eligible for participation. In this paper, only participants with suspected or confirmed COVID-19 were included. We defined suspected COVID-19 as a diagnosis set by a doctor with or without a confirmatory PCR test, reported by the participants. The rationale for including participants without a confirmatory PCR test, thus relying on a clinical diagnosis by a physician alone, was the relatively high prevalence of COVID-19 in the first months of the pandemic combined with the restricted availability of PCR tests. During this period, testing was restricted to healthcare personnel and their close relatives, and in all other cases, the diagnosis was based on clinical assessment. Hospitalized patients were not invited to participate. We recruited participants through different measures. Invitation to participate was communicated through national and local press, in social media channels as well as through our professional network of municipal chief medical officers. In municipalities with ongoing outbreaks, municipal chief medical officers were approached by e-mail and encouraged to inform adult patients with confirmed or suspected COVID-19 about the study. Local health authorities were invited to display study posters in their test centers.

The questionnaire for the online survey was piloted among laypeople and healthcare personnel. Questions regarding possible COVID-19 symptoms were based on available knowledge by March 2020. Participants were asked about the duration of their symptoms and if they had a fever (temperature above 37.8 °C) or not. For the other symptoms listed in the questionnaire (dry cough, wet cough, trouble breathing, chest pain, sore throat, nasal symptoms, fatigue, muscle ache, headache, diarrhea, nausea/vomiting, affected sense of smell, and/or taste), participants were asked to score the severity of each symptom in question. This was done by using a Likert scale ranging from 0 to 3. On this scale, participants scored the degree of affliction where 0 represented “none”, 1 “mild”, 2 “moderate”, and 3 “severe”. After grading the different symptoms, participants had the opportunity to comment on their symptoms or describe additional symptoms. Participants were asked if they regularly were followed up by healthcare personnel in the municipality, and if they sought or received information about COVID-19 from their GP (including out-of-hours services). There were also questions regarding vulnerability and comorbidity based on the WHO report form on COVID-19 available in March 2020 (risk factors and smoking status as listed in Table 1) [9]. To keep confidentiality, all survey answers were stored directly in a secure server at the University of Oslo. All consenting participants have agreed to a registry follow-up of health information 12 months after participation.

**Table 1.** A web-based survey among adult, nonhospitalized COVID-19 patients in Norway. Characteristics of participants (*N* = 477).

Characteristics of participants	Number	Percent
Female gender	324	68
Age		
Mean	41.9 years (SD 13.8)	
Range	18–75 years	
Age groups		
< 40 years	218	46
40–60 years	211	44
> 60 years	48	10
Risk factors		
None	304	64
Pregnancy (incl 6 weeks postpartum)	10	2
Cardiovascular disease	49	10
Pulmonary disease	52	11
Diabetes	16	3
Kidney/liver disease	8	2
Neurological disease	13	3
Cancer	8	2
Immunological disorder	8	2
Other^a^	62	13
Smoking status		
Occasional or regular smokers	48	10
Travelling history (outside of Norway)^b^	24	5
Time of participation		
29 Mar 2020 − 31 Jan 2021	371	78
01 Feb 2021 − 31 May 2021	106	22
Follow-up by healthcare personnel	153	32
Information seeking from primary care		
All primary health care (GP and/or municipal care)	304	64
GP	179	38
Only municipal healthcare	125	26

^a^The most frequent were allergies, rheumatoid disease, chronic fatigue syndrome, and obesity.

^b^In most cases to Spain or Austria.

During analyses, symptoms were clustered according to anatomical regions: upper respiratory (nasal symptoms, sore throat, and affected sense of smell and/or taste), lower respiratory (cough and chest pain), and gastrointestinal symptoms. Generalized symptoms included fatigue and/or muscle ache, headache, and fever. In the questionnaire, fever was listed as a dichotomous variable. Reporting any fever was allocated to a Likert score of 2 for severity. Confirmation of temperature above 37.8 centigrade was allocated to a Likert score of 3. We calculated a total symptom score for each participant as the sum of individual symptom scores. We also compared mean symptom scores of participants before and after 1 February 2021, as the alpha mutation emerged and became dominant from February 2021 in Norway.

Level of statistical significance was set to < 0.05. Exploration of the data showed no skewness of the selected variables. Using independent *t*-tests, groups of symptoms were compared by gender, age groups (under 40 years, 40–60 years, and above 60 years), and smoking status. Correlations between symptom groups were measured by Pearson’s correlation coefficient (r). Correlations could be strong (*r* = 0.50–1), moderate (*r* = 0.30–0.49), or low (*r* < 0.29). Data analyses were performed by IBM SPSS Statistics 27®.

### Ethical approval

The project has been approved by the Regional Ethics Committee (REC South-East D, 120955) and the Data Protection Official of the University of Oslo.

## Results

Of the 477 participants, 379 (79%) reported that their COVID-19 diagnosis had been confirmed by PCR test, while the remaining 98 (21%) were clinically diagnosed by a doctor. Participants without test-confirmed COVID-19 were mostly included in the study in March and April 2020, when testing was only available for healthcare workers and other selected groups. Females represented 324/477 (68%) of the participants, and 90% of all participants were younger than 60 years. In Table 1, the characteristics of the patients are presented. A third of the participants (32%) had organized follow-up of their COVID-19 disease by primary care health personnel, and almost 40% had sought or received information about COVID-19 from a GP (Table 1).

### Symptoms and symptom severity

**Figure 1.**
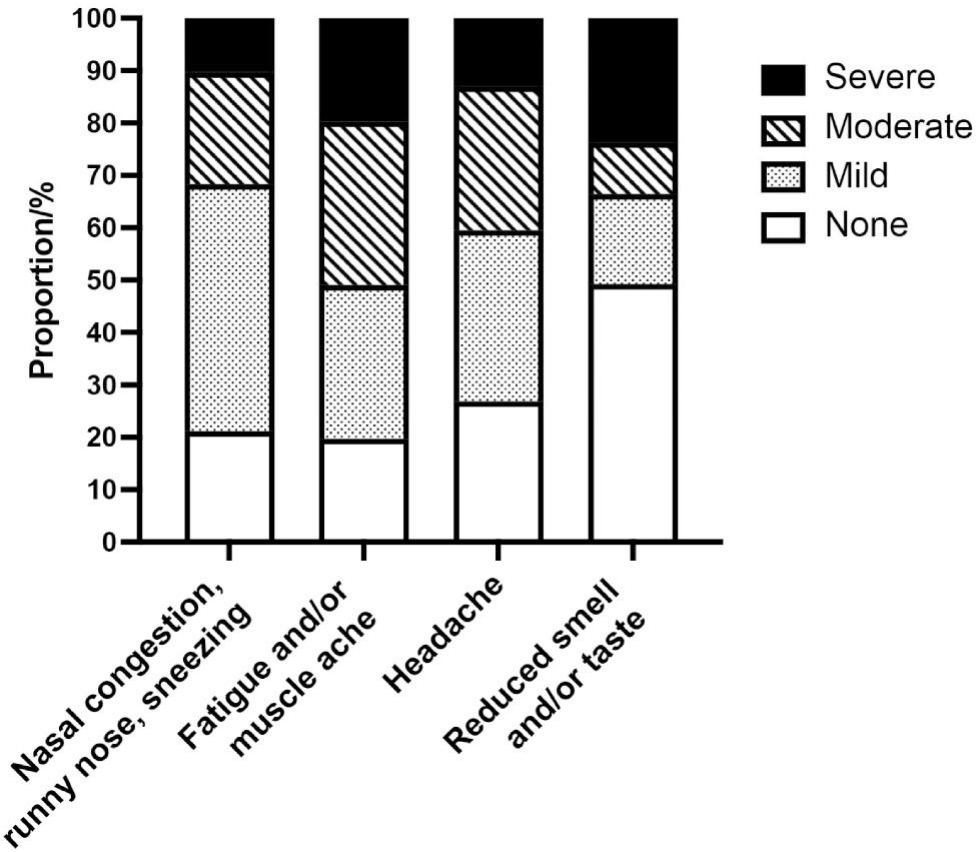
Degree of affliction of the four symptoms with the highest mean symptom score: nasal symptoms, fatigue and/or muscle ache, headache and reduced sense of smell and/or taste. (*N* = 477).

Participants completed the questionnaire at various stages of their COVID-19 illness. A majority (275, 58%) had experienced symptoms for more than 3 days when they were included in the study. Only 25 (5%) answered that they did not have any infection-related symptoms at the time of participation. The mean number of symptoms was 6.4 (SD 2.7). The most common symptoms were “fatigue and/or muscle ache” (in 80% of all), nasal symptoms (79%), and headache (73%). Details of different symptoms affecting the participants are presented in Table 2. The mean scores of symptom severity were generally low on the Likert scale. The symptoms with the highest mean scores were fatigue and headache with mean scores of 1.51 (SD 1.02) and 1.27 (SD 1.00), respectively. However, about half of the participants (234, 49%) reported a maximum symptom score (3 on the Likert scale) for at least one symptom, while 53 (11%) had a score of 3 on the Likert scale for three or more symptoms. The four symptoms where patients reported the highest mean symptom score (highest mean score on the Likert scale) differed in their Likert score distribution (Figure 1). This was most prominent for reduced taste and/or smell, having the lowest mean score of the four (1.08) but the highest proportion of participants scoring 3 (severe affliction) (24%).

**Table 2.** A web-based survey among 477 adult, nonhospitalized COVID-19 patients in Norway. Symptom severity and mean symptom score (Likert (L) score 0-3)*.

Symptom	Mild, moderate or severe affliction (*L* = 1-3)		Severe affliction (*L* = 3)			Mean symptom score^a^ with standard deviation (SD)	
	Number	Percent		Number	Percent	Mean	SD
Fever^b^	178	37		–	–	–	–
Dry cough	331	69		23	5	0.96	0.82
Cough with sputum	239	50		17	4	0.70	0.82
Heavy breathing/shortness of breath	257	54		15	3	0.74	0.81
Chest pain/painful breathing	155	33		16	3	0.49	0.80
Sore throat	287	60		34	7	0.88	0.90
Nasal congestion, runny nose, sneezing	376	79		49	10	1.21	0.89
Fatigue and muscle ache	383	80		94	20	1.51	1.02
Headache	349	73		62	13	1.27	1.00
Diarrhoea	145	30		12	3	0.42	0.73
Nausea/vomiting	99	21		3	1	0.28	0.60
Reduced smell and/or taste	242	51		113	24	1.08	1.24

^a^Likert scale representing the degree of affliction ranging from 0 to 3, where 0 represents “none”, 1 “mild”, 2 “moderate”, and 3 “severe”.

^b^Fever was reported as present or not.

Additional comments or descriptions of symptoms were reported by 159 participants (33%). The most mentioned symptoms in this free text field were back pain (19 participants) and dizziness (16 participants).

### Comparisons for gender, age, follow-up, information seeking, and time period of pandemic

For the different symptoms, as well as total symptom score, the mean scores were slightly higher in female participants compared to the males (Table 3). The mean total symptom scores (maximum 32) were 11.15 (SD 6.02) for females and 9.10 (SD 5.48) for male participants, *p* < 0.001.

**Table 3.** Overall symptom score and groups of related symptoms by gender, age group, smoking status and time period during the pandemic. A web-based survey among 477 adult, nonhospitalized COVID-19 patients in Norway. Figures are means with standard deviations (SD) if not stated otherwise.

	Overall symptom score		Groups of related symptoms			
	Mean total symptom score (SD)	Mean number of symptoms	General symptoms (fever, fatigue, headache), mean	Upper respiratory symptoms, mean	Lower respiratory symptoms, mean	Gastrointestinal symptoms
	(Range 0–32)	(Range 0–12)	(Range 0–3)	(Range 0 –3)	(Range 0–3)	(Range 0–3)
Overall	10.49 (5.93)	6.38 (2.67)	1.24 (0.83)	1.06 (0.68)	0.72 (0.58)	0.35 (0.53)
Gender						
Female	11.15 (6.02)	6.58 (2.61)	1.33 (0.83)	1.13 (0.68)	0.74 (0.59)	0.40 (0.58)
Male	9.10 (5.48)	5.93 (2.75)	1.05 (0.78)	0.90 (0.66)	0.69 (0.55)	0.25 (0.41)
′Mean diff*	**2.04 (0.91 − 3.17)**	**0.65 (0.14 − 1.16)**	**0.28 (0.13 − 0.44)**	**0.23 (0.10 − 0.35)**	0.05 (−0.06 to 0.16)	**0.15 (0.05 − 0.26)**
Age group						
	10.72 (6.14)	6.46 (2.76)	1.20 (0.83)	**1.15 (0.69)^a^**	0.73 (0.58)	0.37 (0.57)
40–60 years	10.31 (5.81)	6.31 (2.64)	1.29 (0.82)	**0.97 (0.65)^a^**	0.71 (0.59)	0.34 (0.51)
> 60 years	10.25 (5.49)	6.25 (2.43)	1.24 (0.83)	0.99 (0.71)	0.72 (0.56)	0.34 (0.49)
Time of pandemic						
< 01.02.2021	10.71 (5.97)	6.46 (2.63)	1.25 (0.84)	1.07 (0.68)	0.75 (0.60)	0.37 (0.54)
> 01.02.2021	9.74 (5.71)	6.08 (2.79)	1.20 (0.79)	1.00 (0.67)	0.64 (0.51)	0.29 (0.52)
Smoking status						
Nonsmoker	10.34 (5.85)	6.30 (2.86)	1.23 (0.82)	1.05 (0.68)	0.71 (0.57)	0.34 (0.53)
Smoker	11.67 (6.42)	6.98 (2.65)	1.35 (0.90)	1.13 (0.68)	0.84 (0.61)	0.43 (0.56)
Follow-up primary care						
Yes	11.19 (6.56)	6.46 (2.71)	1.35 (0.90)	1.12 (0.71)	0.75 (0.63)	0.38 (0.61)
No	10.16 (5.58)	6.33 (2.65)	1.19 (0.79)	1.03 (0.66)	0.71 (0.56)	0.34 (0.49)
Information from GP						
Yes	11.39 (6.36)	6.66 (2.80)	1.31 (0.86)	1.12 (0.70)	0.83 (0.62)	0.40 (0.58)
No	9.95 (5.59)	6.20 (2.58)	1.20 (0.81)	1.02 (0.67)	0.66 (0.55)	0.33 (0.50)
Mean diff^b^	**1.44 (0.35 − 2.54)**	0.46 (-0.95 − 0.04)	0.10 (-0.26 − 0.05)	0.09 (-0.22 − 0.03)	**0.18 (0.07 − 0.28)**	0.07 (-0.17 − 0.03)

^a^ Difference between age groups < 40 years and 40–60 years.

^b^ Difference between means. 95% confidence intervals are shown in brackets, significant differences in bold.

The individual symptom scores did not vary significantly with age (Table 3). However, for upper respiratory tract symptoms as a group, participants aged under 40 years (mean 1.15) had a higher symptom score than participants aged 40–60 years (mean 0.97), *p* = 0.005 (Table 3).

The total symptom score as well as the regional symptom scores did not vary whether the patients reported follow-up by primary care health personnel or not (Table 3). The total symptom score was slightly higher in patients who reported seeking and/or receiving information about COVID-19 from a GP, with a mean symptom score of 11.39 (SD 6.36), compared to a mean total symptom score of 9.95 (SD 5.59) in patients seeking and/or receiving information from other sources. A similar pattern was seen for patients with the highest symptom scores for lower respiratory tract symptoms.

General symptoms and lower respiratory symptoms were positively correlated (*r* = 0.49, *p* < 0.001), while a more moderate, however significant, correlation was seen between upper and lower respiratory symptoms (*r* = 0.35, *p* < 0.001). No significant differences were found in the total mean symptom scores between smokers/nonsmokers or in groups of symptoms between early and later time periods of the pandemic.

## Discussion

### Summary

The mean severity of symptoms among nonhospitalized adult Norwegian COVID-19 patients was generally low. Mean Likert scale scores for degree of severity ranged from 0.28 (nausea) to 1.51 (fatigue and/or muscle ache). Women reported higher symptom scores than men. We found large variations in the occurrence and severity of the symptoms. The most common symptoms were fatigue and/or muscle ache, nasal symptoms, and headache, but the symptom where most patients were severely afflicted was a reduced sense of smell and/or taste. Patients with a higher total symptom score and higher symptom scores from the lower respiratory tract were more likely to seek and/or receive information about COVID-19 from a GP.

### Strengths and limitations

Only a limited number of studies have been conducted in primary care, compared to hospital settings during the pandemic. We consider it a strength that our study only included nonhospitalized patients, as most COVID-19 patients never needed hospitalization. Another strength of this study is that the participants graded the severity of their symptoms. Few other studies from primary care have graded individual symptoms. Our study had a satisfactory age distribution among adult participants, considering the age distribution of patients in the country at the time. Others have reported variations in symptom distribution depending on the time period during the pandemic (with different virus variants dominating) [10]. We recruited participants during more than 1 year of the pandemic, possibly leading to the inclusion of participants infected with different virus strains.

One limitation of our study is the lack of statistical power to do further subgroup analyses particularly within age groups, exemplified by a limited number of participants over 60 years (less than 50 individuals). Another limitation is that one in five participants did not have laboratory-confirmed COVID-19. However, almost all were from the period with strictly restricted testing, when testing was only available for selected groups of patients and health personnel. We consider it likely that these participants also had COVID-19, as the survey asked for COVID-19 to be diagnosed by a doctor, and the prevalence of COVID-19 was high in Norway at that time. These participants were comparable with other participants with confirmed PCR test for most symptom scores. The participants in the study were unselected. However, it is possible that people experiencing symptoms were more likely to participate. We have an overweight of female participants; however, this is not uncommon for web surveys. Our study was not designed for estimating the proportion of asymptomatic participants with confirmed COVID-19. Only 22% of the participants participated in the survey after 1 February 2021, limiting the number of participants possibly infected with later occurring SARS-CoV-2 variants as well as the number of vaccinated participants.

### Comparison with existing literature

In a systematic review from 2022 on signs and symptoms of COVID-19, Struyf et al. [5] reported cough and fever to have the highest sensitivity, whereas all tested symptoms except anosmia (loss of smell) and ageusia (loss of taste) had low specificity. In a large Italian web-based survey from 2020, Adorni et al. [11] showed that COVID-19-positive nonhospitalized participants reported myalgia (61.6%), affected sense of smell and/or taste (59.2%), cough (54.4%), and fever (51.9%) as the most frequent symptoms. Our results showed similar numbers for fatigue and/or muscle ache (80%), affected sense of smell and/or taste (51%) and cough (69% had dry cough). However, only 37% of the participants reported fever in our study. The proportion of asymptomatic individuals in our study was 5%, which is similar to the results reported by Adorni et al. (7.7%) [11]. A review of studies published up to November 2020, before the vaccination programs and the spread of new mutations, found that one-third of SARS-CoV-2 infections were asymptomatic, often throughout the whole duration of laboratory and clinical monitoring [12].

Grading of symptoms for RTIs is commonly used in observational studies and in randomized controlled trials [13–15]. We found large variations in the occurrence and severity of the same symptom between patients. This was most pronounced for “affected sense of smell and/or taste”, where about half of the patients did not experience any smell or taste dysfunction at all, while one in four reported being severely afflicted by the same symptom. In a review, Boscutti et al. [16] referred to primary care studies detecting this complaint in 0-98% of patients. A study of European COVID-19 patients with mild-to-moderate disease found that 85% of the patients had an affected sense of smell. Among these, around 80% reported a total loss of smell [17]. In comparison, only 10% of patients experiencing nasal congestion, runny nose and sneezing reported a high symptom burden. Cough, both dry and with sputum, had a pattern comparable to nasal symptoms. This was similar to the findings in studies of other RTIs, where most patients had mild symptoms independent of etiology [14]. For the systemic symptoms of fatigue/muscle ache and headache, the symptom scores in our study were more evenly distributed. The severity of symptoms during the acute infection may predict the occurrence of persisting symptoms [18,19]. A study from Israeli general practice found that long-COVID symptoms like decreased smell and taste sensation, memory disturbances, dyspnea and arthralgia were frequently seen following a mild symptomatic COVID-19 infection and, to a lesser extent, following an asymptomatic SARS-CoV-2 infection [20].

For all symptoms, as well as for the total symptom score, female participants had a slightly higher symptom severity compared to male participants. Other studies have found that women are more likely than men to report most COVID-19 symptoms [10] and that women also tend to experience more symptoms than men [21]. The same gender difference in symptom reporting has been reported for influenza [22]. However, male patients have a higher risk of a more serious acute course of COVID-19, including higher risk of all-cause death and admission to intensive care units [23]. We did not find a difference in symptom score between age groups. Other studies including older age groups have found marked variation in individual symptoms across age, with children and elderly adults reporting less of the typical symptoms than younger adults [10,24]. Old age is associated with more severe disease and higher mortality with COVID-19 [25]. We suspect that in our study, lack of statistical power may be a reason for our results.

GPs reorganized their clinical work during the pandemic with alternative consultation forms including increased use of video consultations [7,26,27]. The number of face-to-face in-clinic visits declined before the introduction of telehealth consultations led to an increase in total general practice contacts in Denmark [28] and a maintained total visit volume in Norway and Sweden [29]. Our study supports that patients and doctors in primary care prioritized doctor–patient contacts in cases with more symptoms and symptoms from the lower respiratory tract, thus focusing on more severely ill patients. Being able to improvize and accept alternative patient assessments while maintaining adequate health care services was highlighted by Norwegian GPs in our interview study [7]. Primary care needs tailored guidance as early as possible in a health crisis to support clinicians in managing the competing demands of responding to emergency situations while keeping up with usual care [30].

### Conclusions and implications for research and practice

COVID-19 joins the rank of respiratory infectious diseases commonly encountered in primary care. The mean severity of symptoms among nonhospitalized adult COVID-19 patients was generally low. Throughout the pandemic, attention has been directed toward differentiating between symptoms rather than symptom management, resulting in increased focus on disease etiology. Our study shows how GPs appear to prioritize patients seeming more severely ill, even in a challenging situation with a new and unknown disease. We therefore hope primary care can withstand an increased focus on testing and keep trust in the GPs’ clinical judgment. Our study shows the benefit of reporting the intensity of symptoms, as the symptom severity varies for individual symptoms. We encourage other researchers to add this aspect when designing surveys for patients with various infectious diseases. Different symptoms seem to affect some patients more than others. Smell and/or taste dysfunction are symptoms patients reported as bothersome, while nasal congestion and runny nose were symptoms not perceived as equally troublesome. Registry follow-up of our cohort may show if this continues to be a significant problem for patients.
